# BRCA1 and BRCA2 as prognostic markers in oral squamous cell carcinoma: a minireview

**DOI:** 10.3389/fonc.2025.1528822

**Published:** 2025-03-28

**Authors:** Dominika Gedeonová, Claretta Bianchi, Jan Štembírek, Matouš Hrdinka, Zuzana Chyra, Marcela Buchtová, Pavel Hurník, Tomáš Blažek, Jana Režnarová

**Affiliations:** ^1^ Department of Oral and Maxillofacial Surgery, University Hospital Ostrava, Ostrava, Czechia; ^2^ Department of Craniofacial Surgery, Faculty of Medicine, University of Ostrava, Ostrava, Czechia; ^3^ Health Research Centre, Faculty of Medicine, University of Ostrava, Ostrava, Czechia; ^4^ Institute of Animal Physiology and Genetics, Czech Academy of Sciences, Brno, Czechia; ^5^ Department of Hematooncology, University Hospital Ostrava, Ostrava, Czechia; ^6^ Department of Hematology, Faculty of Medicine, University of Ostrava, Ostrava, Czechia; ^7^ Department of Experimental Biology, Faculty of Science, Masaryk University, Brno, Czechia; ^8^ Institute of Molecular and Clinical Pathology and Medical Genetics, University Hospital Ostrava, Ostrava, Czechia; ^9^ Institute of Molecular and Clinical Pathology and Medical Genetics, Faculty of Medicine, University of Ostrava, Ostrava, Czechia; ^10^ Clinic of Oncology, University Hospital Ostrava, Ostrava, Czechia

**Keywords:** BRCA1, BRCA2, OSCC (oral squamous cell carcinoma), HNSCC (head and neck squamous cell carcinoma), gene alteration

## Abstract

Oral squamous cell carcinoma (OSCC), a subset of head and neck cancers, primarily originates in the epithelial tissues of the oral cavity. Despite advancements in treatment, the mortality rate for OSCC remains around 50%, underscoring the urgent need for improved prognostic markers. This review explores the role of the *BRCA1* and *BRCA2* genes—traditionally associated with breast and ovarian cancers—in the context of OSCC. We discuss the molecular pathways involving *BRCA* genes, their potential as diagnostics and prognostic biomarkers, and their implications for personalized treatment strategies, including addressing chemotherapy resistance. Furthermore, this review emphasizes the significance of genome stability in cancer progression and examines both current and emerging methodologies for detecting *BRCA* mutations in OSCC patients. Despite limited prevalence of BRCA mutations in OSCC compared to other cancers, their role in DNA repair and therapeutic response underscores their potential as clinical biomarkers. However, standardized, multicenter studies are still needed to validate their utility in OSCC management. A better understanding of the role of *BRCA* genes in OSCC could pave the way for more effective therapeutic approaches and improved patient outcomes.

## Introduction

**Figure 1:**
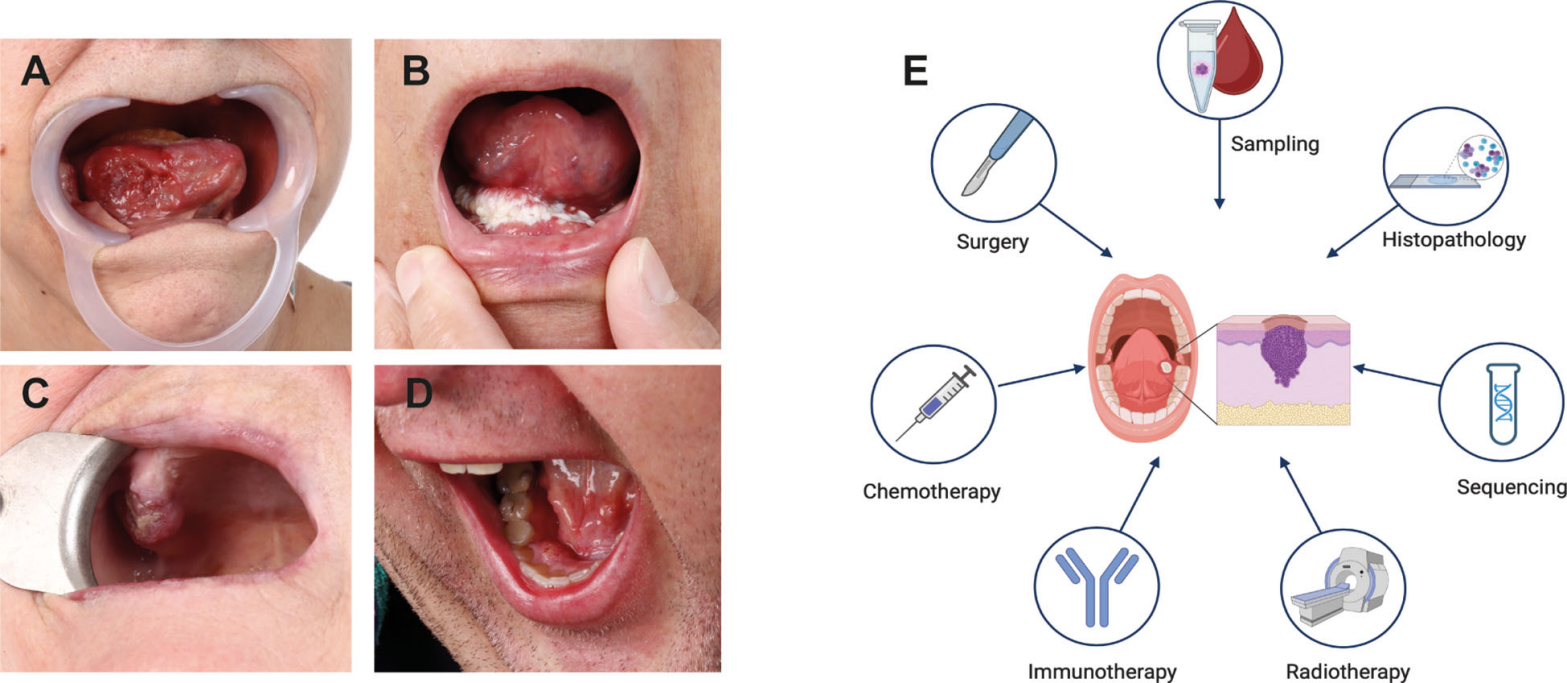
OSCC Tumor Location and Treatment Modalities. **(A-D)** Most common sites of OSCC occur in the oral cavity. **(A)** OSCC growing endophytically on the right side of the tongue. **(B)** OSCC of the floor of the oral cavity extending to the tongue and the mucosa of the alveolar ridge on the right. **(C)** OSCC affecting the alveolar process of the upper jaw on the right side. **(D)** OSCC located on the lingual side of the lower jaw’s alveolar process on the right side. **(E)** Schematic representation of commonly used OSCC treatment strategies. Created in https://BioRender.com.

Oral squamous cell carcinoma (OSCC), a subtype of Head and Neck Squamous Cell Carcinoma (HNSCC), typically originates in the epithelial tissue of the gingiva, tongue, buccal mucosa, palate, and oral floor (1). While OSCC ranks as the sixteenth most common cancer globally, it is the second most widespread in certain high-risk regions (e.g., South Asia) (2), particularly due to the consumption of carcinogen-containing products (3). In contrast, the increasing incidence of oropharyngeal squamous cell carcinomas in Western countries, including the USA, has been linked to an increase in oropharyngeal human papillomavirus (HPV) infection (4). OSCC arises from multifactorial interactions between genetic mutations, environmental exposures, and immune dysregulation, making personalized treatment approaches particularly challenging (5) (**Figures 1A–D**). The progression to invasive OSCC involves a series of cellular changes, beginning with epithelial hyperplasia, progressing through various grades of dysplasia, and culminating in invasive carcinoma. These changes are driven by genomic alterations (6), which disrupt the balance between oncogenic and suppressor signaling pathways (5).

Oncogenic pathways, such as EGFR, PI3K/AKT/mTOR, JAK/STAT, MET, Wnt/β-catenin, and RAS/RAF/MAPK, are often abnormally activated in OSCC, while tumor suppressor pathways like TP53/RB, p16/Cyclin D1/Rb, and NOTCH are frequently inactivated (5).

Among these genomic alterations, BRCA1 and BRCA2—genes traditionally associated with breast and ovarian cancers—are emerging as key players in OSCC due to their roles in maintaining genomic stability and regulating DNA damage repair. This review explores the potential of *BRCA1* and *BRCA2* as prognostic markers in OSCC by elucidating their involvement in molecular pathways and their diagnostic or therapeutic implications. We also aim to provide insights into their significance in OSCC management and patient outcomes.

## BRCA genes and their role in genome stability

**Figure 2:**
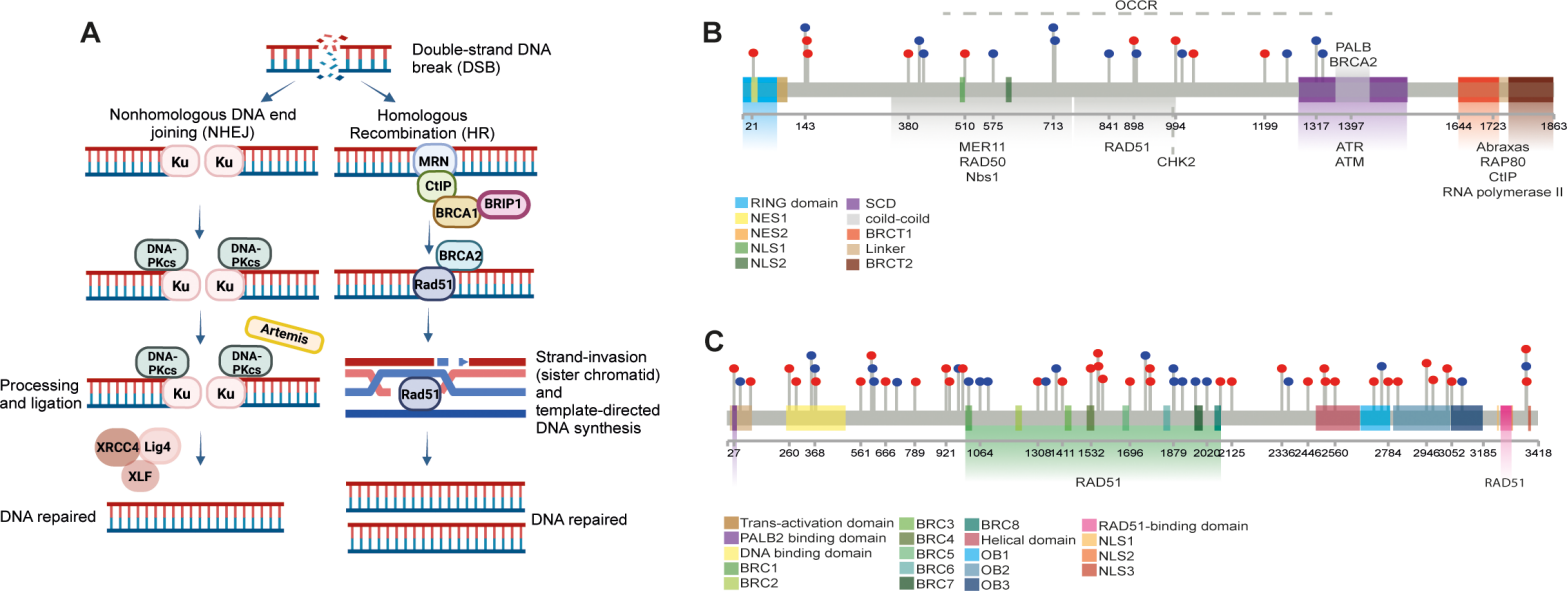
Overview of DNA Damage Response (DDR) and BRCA1/2 Functions in OSCC. **(A)** Schematic illustration of the DNA damage response (DDR) pathways, highlighting the roles of non-homologous (NHEJ) and homologous recombination (HR) in repairing double-strand breaks (DSBs). The involvement of BRCA1 and BRCA2 in HR-mediated repair of damaged DNA is depicted. Created at https://BioRender.com. **(B, C)** Lollipop schematic diagrams of BRCA1 and BRCA2 proteins, illustrating their structural domains and interactions with other HR-related proteins and enzymes necessary for DNA repair. Single-point mutations in BRCA1 and BRCA2 genes retrieved from online databases (COSMIC, cBioPortal, dbSNP, ClinVar), are indicated by lollipop markers.

Maintaining genome stability is crucial for cellular survival, necessitating an effective DNA damage response (DDR). DDR mechanisms repair double-strand breaks (DSBs) through two primary mechanisms: non-homologous end-joining (NHEJ) and homologous recombination (HR) (7) (**Figure 2A**).

The BRCA proteins–BRCA1 and BRCA2–play integral roles in HR-mediated DSB repair (8). The BRCA1 is characterized by two structural domains: the zinc-binding RING domain at the N-terminus and two phosphopeptide-binding BRCT domains at the C-terminus, facilitating complex protein interactions necessary for DNA repair (9). The RING domain, in combination with BRCA1-associated RING domain protein 1 (BARD1), forms a heterodimer that exhibits E3-ubiquitin ligase activity, essential for tagging damaged DNA and proteins for repair processes (10–12) (**Figure 2B**).

Similarly, BRCA2 contains several functional domains that interact with RAD51 recombinase, crucial for mediating the HR process. For instance, the N-terminal domain interacts with PALB2, a key scaffolding protein in the BRCA complex (13, 14); the central part of BRCA2 contains eight BRC repeats essential for binding RAD51 (15); and the C-terminus mediates DNA interactions via the DNA binding domain (DBD), which contributes to the initiation and stabilization of the repair process (16) (**Figure 2C**).

The HR pathway of DDR employs various proteins to accurately repair DNA without introducing mutations. Initial sensors recognize the break, and signaling mediators engage effectors to restore the damage (**Figure 2A**). Proteins such as ATM and ATR are the first to detect DNA disruption and initiate repair mechanisms. BRCA1 is recruited to DSBs through the Abraxas–RAP80 protein complex (17) and interacts with the MRN complex (MRE11-RAD50-NBS1), facilitating the recruitment of CtIP protein, which catalyzes DNA end resection alongside exonuclease EXOI (18).

BRCA1 also plays a central role in facilitating BRCA2 recruitment to DSBs, subsequently binds RAD51 and initiates HR on the single-stranded DNA (ssDNA) (19). The BRCA1-interacting protein 1 (BRIP1) binds to BRCA1 at the BRCT domain, unwinding DNA during HR and enabling the recruitment of RAD51 (20). This process begins with the formation of a D-loop DNA structure by RAD51, enabling template-directed DNA synthesis (21). Polymerase δ extends the invading strand, and DNA ligase seals the strand breaks, fully restoring DNA integrity (22).

In addition to DSB repair, genome stability is also compromised by interstrand crosslinks (ICLs). The Fanconi anemia (FA) pathway plays a crucial role in repairing ICLs. At least 19 proteins are involved in this pathway, including BRCA1 (FANCS), BRCA2 (FANCD1), RAD51 (FANCR) and BRIP1 (FANCJ). The FA pathway core complex recognizes ICLs and facilitates the monoubiquitination of Fanconi anemia group D2 protein (FANCD2), which is essential for activating the pathway. The monoubiquitinated FANCD2 interacts with BRCA1, allowing the recruitment of HR repair machinery, including BRCA2 and RAD51. BRIP1 is also recruited to unwind the DNA and support the repair process, resolving crosslinks and enabling the DNA repair proteins to access the damage (23).

In the context of OSCC, disruptions in BRCA1 and BRCA2 due to mutations or dysregulations significantly impair the HR and FA pathways, leading to genomic instability and contributing to carcinogenesis. This underscores the importance of BRCA1 and BRCA2 not only in hereditary cancers but also in the pathophysiology of OSCC, positioning them as potential therapeutic targets and valuable prognostic biomarkers (24).

## The role of BRCA genes in OSCC tumorigenesis

The role of *BRCA* genes in maintaining genomic integrity suggests their potential significance in multiple cancers, including OSCC. Recent research on BRCA gene alterations in OSCC has employed various approaches, including proteomic, genomic, and differential gene expression studies. Immunohistochemistry (IHC) is commonly used to examine BRCA protein distribution in tumorous tissues, while quantitative PCR (qPCR) evaluates differential gene expression (25–27). However, more precise sequencing methods are needed to identify specific gene mutations, which could provide deeper insights into the genetic underpinnings of OSCC (28–30).

Several studies have investigated the role of BRCA proteins in the transformation of oral leukoplakia (OLK) to OSCC. Vora et al. (31) conducted a detailed evaluation of BRCA1 expression in specimens from 77 patients with early-stage and locally advanced SCC of the tongue and 18 patients with leukoplakia of the tongue, utilizing IHC techniques with a semi-quantitative staining intensity score ranging from negative (no staining) to 3+ (31). Their findings indicated that BRCA1 protein levels were higher in OLK compared to OSCC, suggesting potential downregulation during tumorigenesis. Among OLK samples with a staining intensity of 2+, hyperplastic tissues exhibited lower BRCA1 expression compared to dysplastic tissues. The distribution of BRCA1 in all evaluated tissues was predominantly cytoplasmic, and the protein was completely absent in 66% of OSCC samples, suggesting that the loss of BRCA1 function is relevant to neoplastic transformation. Vora et al. further noted that the observed cytoplasmic staining of BRCA1 may be attributed to naturally occurring alternatively spliced variants of BRCA1 that lack most of the exon 11 sequences and do not possess a nuclear localization signal. These cytoplasmic variants were prevalent in OSCC tissues. Interestingly, among patients with a family history of cancer, 63% expressed BRCA1 were with 1+ (80%) and 2+ (20%) staining intensities. In early-stage disease, BRCA1-positive patients exhibited reduced relapse-free survival compared to BRCA1-negative patients. However, no correlation was observed between clinicopathological parameters and BRCA1 expression. Additionally, a positive correlation between BRCA1 and *c-myc* expression, used as a predictor of unfavorable prognosis, was identified, further supporting the dynamic role of BRCA1 across different stages of OSCC development. These findings underscore the need for further research to validate BRCA1 as a prognostic marker.

BRCA1 and γH2AX were proposed as independent prognosis markers of OSCC by Oliveira-Costa et al. (2014), who evaluated protein and RNA levels (25). γH2AX,a phosphorylated histone protein correlated with DNA damage (32), was positively associated with poor overall survival. Interestingly, BRCA1 was detected predominantly in the cytoplasm, an unusual localization under conditions of cellular stress induced by DNA damage. These findings emphasize the need for further investigation into BRCA1 expression patterns and their clinical significance. Cytoplasmic localization of BRCA1/2, which typically reflects impaired functionality, has been documented in various cancers, including breast, prostate, gastric, colorectal, and pancreatic cancers (33–35). BRCA1 mutations in the BRCT domain are known to alter its nuclear localization, driving cytoplasmic retention and loss of nuclear repair functionality (36, 37). Chen et al. (38) demonstrated that BRCA1 was entirely mislocalized to the cytoplasm in breast cancer cells (39). Interestingly, cytoplasmic BRCA1/2 expression has been linked to better prognosis in breast and gastric cancers but is less explored in OSCC (33, 40).

A more recent study by Irani and Rafidazeh (2020) further explored the expression profiles of BRCA1 and BRCA2 in OSCC through retrospective analysis (26). Their findings indicated that 63.3% of intermediate and high-grade OSCC tissues exhibited moderate to strong cytoplasmic BRCA1 immunoreactivity, while only 28.3% displayed nuclear BRCA1 expression. Similarly, BRCA2 expression was entirely cytoplasmic, with 55.01% of intermediate and high-grade tissues showing moderate to strong cytoplasmic immunoreactivity. Only four low-grade samples (two for each protein) displayed strong cytoplasmic BRCA1/2 expression. Importantly, BRCA1 was also detected at the invasive front and in the detached tumor cells, suggesting its possible involvement in epithelial-mesenchymal transition (EMT) processes via regulation of E-cadherin and vimentin levels (41). These findings suggest that the aberrant subcellular localization of BRCA1/2, along with their expression at the invasive front, plays an essential role in OSCC pathogenesis and could serve as prognostic markers.

Despite these promising findings, variability across studies raises questions about methodological differences. Specifically, Vora et al. (31) used an antibody targeting the N-terminal region of BRCA1 (clone MS13), while Irani and Rafidazeh employed the MS110 antibody, whose immunogen was undisclosed (26, 31). Interestingly, Oliveira-Costa et al. used the same antibody as Vora et al. but reported findings more consistent with those of Irani and Rafidazeh. This suggests that factors beyond antibody selection, such as sample size, patient demographics, and study design, may also contribute to observed differences. Additionally, the choice of experimental endpoints, such as whether cytoplasmic versus nuclear localization is prioritized, may further influence conclusions across studies. Standardized methodologies and larger cohorts will be critical to reconciling these discrepancies.

Beyond their subcellular localization, genomic studies have provided further insights into BRCA1/2 mutations in OSCC pathogenesis. Exome sequencing of OLK and OSCC has identified BRCA1 and BRCA2 as key markers distinguishing progressive from non-progressive lesions (42). Using multivariate analysis, researchers demonstrated that BRCA1/2 expression decreases with lesion severity, from normal tissue to OSCC. Interestingly, these findings contrast with earlier studies, possibly due to differences in methodology.

High-resolution array-based comparative genomic hybridization (aCGH) has revealed frequent mutations in DDR pathway genes, including BRCA1, BRCA2, FANCD2, and FANCG, in over 25% of OSCC samples (43). Amplifications in BRCA1 and FANCG were observed in 33% and 29% of samples, respectively, while deletions in BRCA2 and FANCD2 occurred in 38% and 33% of samples. These findings highlight the critical role of the FA/BRCA pathway, which is central to DDR, in OSCC pathogenesis. BRCA1 (FANCS) and BRCA2 (FANCD1) are integral components of this pathway, which is responsible for repairing DNA interstrand crosslinks and maintaining genomic stability.

Disruptions in the FA/BRCA pathway, such as BRCA1/2 mutations, impair DNA damage repair processes, leading to increased genomic instability and heightened susceptibility to malignancies, including OSCC. Supporting this, studies on Fanconi anemia (FA)—a genetic disorder caused by mutations in one of 22 genes involved in the FA pathway—highlight a strong connection between FA gene dysfunction and OSCC. FA patients frequently develop OSCC, underscoring the importance of BRCA1/2 and other FA pathway genes in the disease’s pathogenesis (44). The high prevalence of OSCC in FA patients provides indirect evidence that alterations in BRCA1/2 contribute to tumorigenesis in non-FA populations as well. These findings reinforce the potential of BRCA genes as biomarkers for identifying genomic instability in OSCC and highlight their importance as therapeutic targets in this malignancy.

## Discussion

Oral squamous cell carcinoma (OSCC) develops through a complex interplay of genetic and environmental factors, contributing to its aggressive nature and genomic instability. Identifying metabolic, molecular, and immune characteristics that serve as predictive and prognostic markers remains a significant challenge. Despite advances in therapy and research, the overall survival rate for OSCC patients has stagnated at around 50%, primarily due to challenges such as late-stage diagnosis, limited availability of reliable biomarkers, and resistance to conventional therapies (25, 45–47). This highlights the urgent need for more effective biomarkers and therapeutic targets to improve early detection, prognostication, and treatment strategies.

*BRCA1* and *BRCA2* genes are well-known for their critical roles in DDR, particularly in HR, a highly accurate DNA repair mechanism (48). While mutations in these genes have been extensively studied in breast and ovarian cancers (49), their significance in other malignancies, including OSCC, is an emerging area of research. Alterations in BRCA gene expression, even in the absence of frequent mutations, have been implicated in OSCC tumorigenesis. Recent studies suggest that BRCA1 and BRCA2 expression patterns vary with tumor grade, potentially reflecting their roles in tumor progression (25–27). However, precise data on the frequency of BRCA mutations in OSCC remain limited. While somatic BRCA mutations in breast and ovarian cancers range between 5–20% (47, 50, 51), and pathogenic BRCA2 mutations occur in 2% of pancreatic cancers (52), mutations in OSCC or HNSCC are believed to be rare (25, 26, 28–31, 43, 53). The rarity of BRCA mutations in OSCC may reflect differences in tissue-specific genetic instability and environmental exposures that shape the mutational landscape (51).

Despite the low prevalence of BRCA mutations in OSCC, their altered expression and functional roles in DNA repair highlight their potential as biomarkers. Genomic profiling of OSCC samples has identified occasional *BRCA1* and *BRCA2* mutations, suggesting their contributions to tumor onset and progression (28–30).

Studies on oral leukoplakia (OLK) further suggest that BRCA1 and BRCA2 expression levels may distinguish high-risk dysplastic lesions that progress to carcinoma from those that remain benign (42, 54). Thus, assessing BRCA1/2 expression using standardized IHC scoring could help stratify patients based on their progression risk, enabling more targeted interventions. These findings underscore the potential utility of *BRCA* genes as early biomarkers in OPMD, offering an opportunity to identify patients at greatest risk of developing OSCC and enabling timely intervention.

The functions of *BRCA1* and *BRCA2* in DNA repair and cell cycle regulation are of particular interest because defects in these processes lead to genomic instability, a hallmark of carcinogenesis. Proper cell cycle regulation and DNA repair are essential for maintaining the integrity of the oral epithelium, which is highly susceptible to mutagenic damage from external factors such as tobacco, alcohol, and HPV infection, as well as internal cellular processes (5, 55). Studies suggest that DDR processes, including BRCA1 activity, are activated during the early stages of OSCC to mitigate genomic instability and support tumor survival. Increased expression of BRCA1 and other DNA repair proteins, such as p53, γ-H2AX, RAD51, and 53BP1, has been observed in early OSCC development, highlighting the potential of these molecules, particularly BRCA1, as prognostic biomarkers (25, 55, 56). The potential involvement of BRCA genes in treatment response has also gained attention. Standard care for locally advanced OSCC typically includes surgery followed by adjuvant radiotherapy, with or without chemotherapy (57) (**Figure 1E**). However, treatment resistance remains a significant obstacle (58). Molecular targeted therapies using specific antibodies against epidermal growth factor receptor (EGFR) and programmed cell death protein 1 (PD-1) have shown promise (59). BRCA1 expression has been implicated in modulating chemotherapy-induced DNA damage, influencing responses to chemotherapeutic agents such as cisplatin and paclitaxel (60, 61). Increased BRCA1 expression may enhance DNA repair, reducing sensitivity to DNA-damaging agents like cisplatin, while simultaneously increasing sensitivity to microtubule-targeting agents such as paclitaxel and docetaxel (62–64). Similarly, in tongue squamous cell carcinoma, the BRCA1-miR-593-5p-MFF axis regulates cisplatin sensitivity by modulating mitochondrial fission and apoptosis, further highlighting the complex role of BRCA1 in therapeutic responses (64).

Like BRCA1, BRCA2 plays a critical role in maintaining genomic stability (**Figures 2B, C**), and its mutations may impair DNA repair pathways, increasing susceptibility to oncogenic mutations in oral tissues (27). This has significant implications for targeted therapies, particularly Poly(ADP-ribose) polymerase (PARP) inhibitors(PARPi). PARPi block the activity of PARP enzymes, which are involved in repairing single-strand DNA breaks (65–67). In cells with BRCA mutations, where HR is dysfunctional, PARP inhibition leads to the accumulation of unrepaired DNA damage, resulting in cell death through synthetic lethality (68, 69). Given that OSCC often exhibits aberrant DNA repair pathways, including nucleotide excision repair (NER), base excision repair (BER), and double-strand break repair (DSBR) (70–73), PARPi represent a promising therapeutic strategy. However, emerging resistance mechanisms, such as secondary mutations restoring homologous recombination, highlight the need for combination strategies to enhance efficacy (74). Incorporating genetic analysis and molecular profiling into standard diagnostic protocols could identify OSCC patients most likely to benefit from PARPi and other targeted therapies, thereby improving outcomes.In summary, BRCA1 and BRCA2 are central to maintaining genomic stability by facilitating the repair of DNA double-strand breaks through homologous recombination. Their mutations or dysregulation in OSCC can disrupt DNA repair mechanisms, leading to genomic instability and oncogenic transformation. While their role in OSCC is not as well-defined as in breast and ovarian cancers, emerging evidence suggests that BRCA1 and BRCA2 contribute to tumor initiation, progression, and therapeutic resistance (25–27, 53, 75). Moreover, their involvement in the Fanconi anemia pathway and interactions with other DDR proteins underscore their significance in complex repair networks. Future studies focusing on the standardization of methodologies, larger cohorts, and detailed molecular analyses are essential to validate BRCA1 and BRCA2 as biomarkers and therapeutic targets in OSCC. Such advances could pave the way for more personalized and effective treatment strategies, ultimately improving patient outcomes.
